# Immunological characteristics of immunogenic cell death genes and malignant progression driving roles of TLR4 in anaplastic thyroid carcinoma

**DOI:** 10.1186/s12885-023-11647-y

**Published:** 2023-11-21

**Authors:** Tong Xu, Chaozhuang Zhu, Feifeng Song, Wanli Zhang, Mengnan Yuan, Zongfu Pan, Ping Huang

**Affiliations:** 1 Center for Clinical Pharmacy, Cancer Center, Department of Pharmacy, Zhejiang Provincial People’s Hospital (Affiliated People’s Hospital), Hangzhou Medical College, 158 Shangtang Road, Xiacheng District, Hangzhou, Zhejiang 310014 China; 2 Zhejiang University of Technology, Hangzhou, Zhejiang China; 3 Key Laboratory of Endocrine Gland Diseases of Zhejiang Province, Hangzhou, Zhejiang 310014 China

**Keywords:** Immunogenic cell death, Anaplastic thyroid carcinoma, Immune characteristics, TLR4, Malignant progression

## Abstract

Anaplastic thyroid carcinoma (ATC) was a rare malignancy featured with the weak immunotherapeutic response. So far, disorders of immunogenic cell death genes (ICDGs) were identified as the driving factors in cancer progression, while their roles in ATC remained poorly clear. Datasets analysis identified that most ICDGs were high expressed in ATC, while DE-ICDGs were located in module c1_112, which was mainly enriched in Toll-like receptor signalings. Subsequently, the ICD score was established to classify ATC samples into the high and low ICD score groups, and function analysis indicated that high ICD score was associated with the immune characteristics. The high ICD score group had higher proportions of specific immune and stromal cells, as well as increased expression of immune checkpoints. Additionally, TLR4, ENTPD1, LY96, CASP1 and PDIA3 were identified as the dynamic signature in the malignant progression of ATC. Notably, TLR4 was significantly upregulated in ATC tissues, associated with poor prognosis. Silence of TLR4 inhibited the proliferation, metastasis and clone formation of ATC cells. Eventually, silence of TLR4 synergistically enhanced paclitaxel-induced proliferation inhibition, apoptosis, CALR exposure and release of ATP. Our findings highlighted that the aberrant expression of TLR4 drove the malignant progression of ATC, which contributed to our understanding of the roles of ICDGs in ATC.

**Supplementary Information:**

The online version contains supplementary material available at 10.1186/s12885-023-11647-y.

## Introduction

Anaplastic thyroid carcinoma (ATC) was a rare malignancy that accounted for 50% of thyroid cancer deaths [1, 2]. ATC progressed rapidly and was characterized by local and distant metastases. Currently, surgical resection was the main clinical treatment, supplemented by radiotherapy and chemotherapy [3–5]. However, the prognosis of ATC remained very poor, with the median survival of patients often less than 6 months [6]. Hence, it was urgent to explore the crucial molecules that driving the malignant progression of ATC and develop new clinical treatment strategies.

In recent years, the increasing popularity of immunotherapy had greatly encouraged researchers to investigate its potential for treating ATC patients [7]. Clinical trials for patients with locally advanced or metastatic ATC have shown that sparazumab treatment resulted in an objective response rate of 19%. However, the median progression-free survival and overall survival were only 1.7 months and 5.9 months, respectively indicating limited efficacy of single-agent immunotherapy for ATC [8]. And a current clinical study of CSF-1R inhibitor combined with PD-1 inhibitor showed remission in only 37% of patients with advanced thyroid cancer [9]. In another study of immunotherapy combined with chemoradiotherapy for ATC patients, a combination of palizumab, docetaxel, and doxorubicin with volume-modulated intensity radiotherapy was performed, but regrettably all patients died within 6 months [10]. These indicated an urgent need for highly effective strategies to increase sensitivity to ATC immunotherapy.

Immunogenic cell death (ICD) was a form of cell death that was triggered by different cell stressors, such as chemotherapy drugs, oncolytic viruses, radiotherapy and photodynamic therapy [11]. This type of cell death was distinguished by the release of damage-associated molecular patterns (DAMPs), including HMGB1, calreticulin (CALR), ATP, and heat shock proteins. These DAMPs played crucial roles in anti-tumor immunity [12]. Emerging evidences suggested that ICD induction of tumor cells and transforming into anti-tumor vaccines could significantly inhibit tumor growth. Kwong et al. showed that paclitaxel (PTX) induced ICD-related DAMPs in ovarian cancer and significant antitumor responses in vivo [13]. And the combination of Trifluridine and Oxaliplatin eliminated type 2 tumor-associated macrophages, leading to higher cytotoxic CD8 + T cell infiltration and activation, thereby inducing ICD to improve curative effect in patients with colorectal cancer [14]. Oleander was found to trigger endoplasmic reticulum stress and induce ICD-mediated immune destruction of breast cancer cells [15]. Therefore, induction of ICD might act crucial roles in improving the efficacy of ATC immunotherapy. However, there was no studies associated with ICD reported in ATC.

Here, we had integrated the datasets from multiple laboratories or array platforms to conduct the largest ATC cohort gene expression studies. To address the challenges of ATC samples collection and the systematic bias between different datasets, efforts could be made to identify the unique and specifically altered ICDGs signature in ATC. We demonstrated significant ICDGs and constructed the gene co-expression network in ATC. Then we established an ICD score evaluation model to score ATC samples and divided them into two groups. The differential expressed genes (DEGs) of high and low ICD score subgroups were identified and analyzed for functional enrichment. Nonetheless, the differences in immune landscapes and immune checkpoint expression between the two subgroups were compared. Further, we identified the signature of 5 ICDGs to reveal crucial signals for driving the malignant progression of ATC. We found that the upregulation of TLR4 significantly promoted the malignant characteristics of ATC, and inhibition of TLR4 induced ICD in combination with paclitaxel. To our knowledge, this was the first time that functions of ICDGs had been studied in ATC. Moreover, our results demonstrated that the disorder of immunogenic cell death genes were crucial driving factors leading to the malignancy of ATC, and the intervention of TLR4 might be beneficial to the immunotherapy of ATC.

## Materials and methods

### Microarray information

**Fig. 1:**
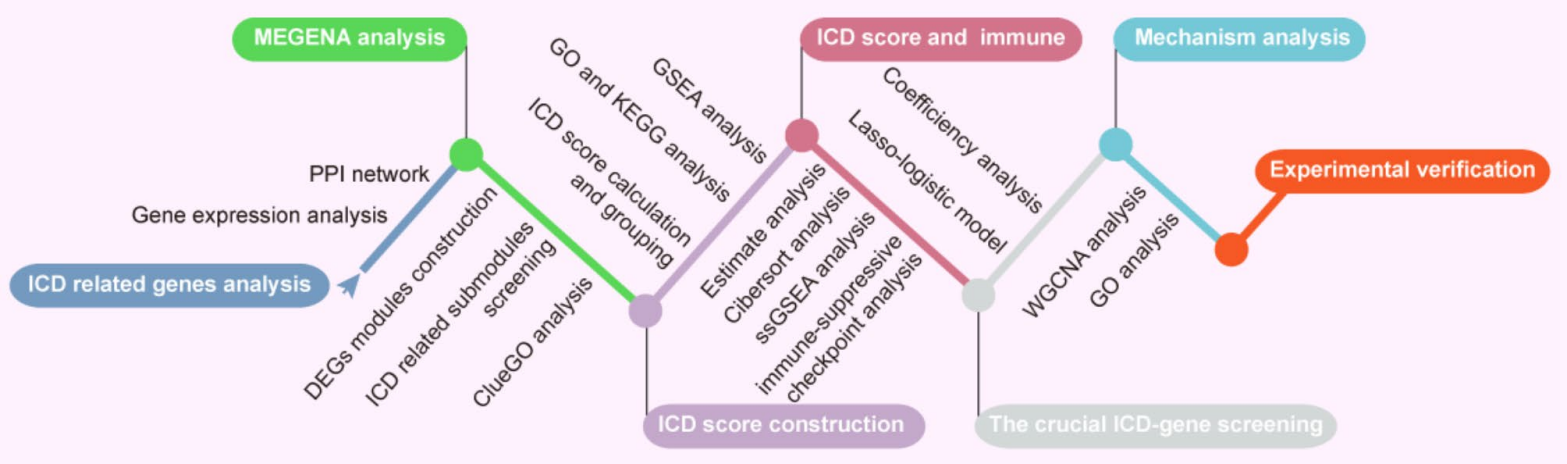
Analysis workflow of this research

The microarray datasets (GSE65144, GSE33630, GSE29265 and GSE76039) in the GEO database (https://www.ncbi.nlm.nih.gov/geo/) were downloaded [16], in which researchers deposit their microarray data related to various diseases. The dataset of GSE65144 included 12 ATCs and 13 normal tissues [17]; GSE33630 contained 11 ATCs, 49 papillary thyroid carcinomas (PTC) and 45 normal tissues [18]; GSE29265 dataset contained 9 ATC, 20 normal tissues and 20 PTCs; GSE76039 had 20 ATCs and 17 poorly-differentiated thyroid carcinomas (PDTCs) [19, 20]. The above datasets were based on GPL570 (Affymetrix HT HG-U133 plus PM Array). The four datasets were corrected and its dimensionality were reduced by the Remove Batch Effect function of the limma package and principal component analysis in the R4.1.3 environment [21, 22]. And analysis workflow of this research was showed in Fig. 1.

### Identification and MEGENA analysis of immunogenic cell death genes

The list of ICDGs was from the meta-analysis of ICD-related literature conducted by Abhishek et al. [23]. The differentially expressed analysis of ATC vs. normal groups were also performed using limma package, and the volcano plot and heatmap plot were made by ggpubr and pheatmap packages respectively. The protein-protein interaction (PPI) network analysis were conducted by the STRING database and optimized by the Cytoscape 3.6.1 software [24]. The ICDGs were analyzed by the Multiscale Embedded Gene co-Expression Network analysis (MEGENA) package for calculating a multi-scale network including different possible variations in gene interactions in R4.1.3 [25]. Then the network enrichment analysis of all node genes of c1_112 was implemented by the ClueGO v2.5.2 plug-in unit of Cytoscape 3.6.1 software.

### The establishment of ICD score model and enrichment analysis

ATC samples in the dataset were scored according to ICDGs expression and ssGSEA algorithm in GSVA package. The mean score of all ATC sample was calculated, and the samples were divided into high ICD score group and low ICD score group based on this. Then the difference genes between the subgroups were identified by the limma package, whose thresholds were log_2_ (fold change) > 1.5 or log_2_ (fold change) < -1. The DEGs was mapped into a pointplot by the ggplot2 package. The GO function annotation, KEGG pathway enrichment and GSEA analysis were performed to further elucidate the biological roles of DEGs between the subgroups by the clusterProfiler package in the R4.1.3 environment.

### Immune characteristics analysis

ESTIMATE, an algorithm that detect the proportion of stromal and immune cells based on gene expression characteristics in tumor samples, was utilized for the calculation of stromal score, immune score, ESTIMATE score and tumor purity of high or low ICD score groups [26]. CIBERSORT that was a deconvolution algorithm was used to calculate the fraction of 22 immune cells in each samples in two subgroups [27]. According to the gene expression of 28 published immune cell genesets combined with ssGSEA algorithm, the differences in the extent of infiltration of 28 immune cell types between the two groups were compared [28].

### The establishment of Lasso-logistic model and prognostic analysis

The Lasso Logistic model by the glmnet package mainly adjusted the λ parameter to complete the variable selection, so that the estimated value of irrelevant variables was 0, and further learned and weighted to predict the ICDGs most related to ATC [29]. The prognostic analysis of ICDGs in thyroid cancers was applied by Kaplan-Meier plotter (https://kmplot.com/analysis/).

### WGCNA analysis

The WGCNA package was used to analyze gene expression patterns in multiple samples [30]. WGCNA was utilized for co-expression analysis of DEGs between high and low TLR4 groups. The mean linkage and Pearson’s correlation methods were used to plot a sample dendrogram with stromal score, immune score, ESTIMATE score and tumor purity. The DEGs were assigned to 6 modules, and 26 key genes of red module were obtained by calculating module membership (MM) and gene significance (GS), followed by GO Enrichment analysis.

### Cell culture and transfection

The 8505 C (Anaplastic thyroid carcinoma cell line) and Nthy-ori 3 − 1 (Normal human thyroid cell line) were purchased from Fenghui Biotechnology. The CAL62 (Anaplastic thyroid carcinoma cell line) was purchased from Procell Life Science&Technology. Cell Line authentication: all cell lines had been performed authenticated using Short Tandem Repeat (STR) analysis on 2021 in Procell Life Science&Technology. The CAL62, 8505 C and Nthy-ori 3 − 1 were cultured in DMEM or RPMI-1640 medium supplemented with 10% FBS (Gbico, USA) and in culture flask (Jet Biofil, TCF012050). 8505 C cell was seeded in 6-well plated with 40–50% confluence, and the siRNAs-TLR4 were transfected with jetPRIME (Polyplus, USA) according to the instruction. The sense sequences of TLR4 siRNA#1 was CAGCAAUCUCACUUCUGUA and antisense sequences was UACAGAAGUG- AGAUUGCUG. The sense sequences of TLR4 siRNA#2 was GUGUGUUUCCAU- GUCUCAU and antisense sequences was AUGAGACAUGGAAACACAC.

### Western blot and qRT-PCR

The operation processes of western blot and qRT-PCR were based on the previous literature published by our team [31]. The primary antibody TLR4 (ABclonal Technology, western blot 1:1000, China) and GAPDH (Proteintech, 1:1000, China) were applied for detection of proteins by the electrophoresis apparatus ( Bio-rad, USA). qRT-PCR primers: The forward primer of TLR4 was 5’-GTGCCTCCATTTCAGCTCTG-3’ and the reverse primer was 5’-CAAAGATAC- ACCAGCGGCTC-3’. The forward primer of β-ACTIN was 5’-ACCTTCTACAATG- AGCTG- CG-3’ and the reverse primer was 5’-CCTGGATAGCAACGTACATGG-3’.

### Immunohistochemistry

The operation processes of immunohistochemistry was based on the previous literature published by our team [31]. The primary antibody TLR4 were applied for immunohistochemistry (TLR4, immunohistochemistry 1:100). The tissue sections ATC1, ATC2 and ATC3 were acquired from the tumor tissue sample bank of Zhejiang Provincial People’s Hospital, which were clinicopathologically diagnosed as anaplastic thyroid carcinoma. The process was approved by the Ethics Committee of Zhejiang Provincial People’s Hospital.

### Cell proliferation, migration, invasion and clone formation

For cell proliferation assay, the treated ATC cells were seeded in the 96-well plate with 30–40% confluence for 48 h growth, and then the viability was detected by the Cell Counting Kit-8 (Fdbio, China) on the microplate reader (Bioteck, USA). For migration assay, the treated ATC cells were seeded in the 12-well plate with 100% confluence replaced with serum-free medium. The cells were scratched at the 0 h and imaged, then observing their migration capacity at 48 h and imaged. For cell invasion assay, the transfected ATC cells were seeded in the upper chamber pre-coated with 5% Matrigel (BD Biosciences, USA). Medium containing 0% and 10% serum were added to the upper and lower chambers for incubating 48 h, respectively. The invasive cells were then fixed with methanol and stained with 0.1% crystal violet (Applygen, China). The infiltrating cells were photographed after the upper chamber cells were gently wiped. For clone formation assay, the treated ATC cells were seeded with 500/12-well to incubate for 1 week, and then cells were fixed with methanol, stained with 0.1% crystal violet (Applygen, China) and photographed.

### Flow cytometry and ATP release detection

For cell apoptosis assay, the treated ATC cells were centrifuged and resuspended with 1X binding buffer (Beyotime, China). Annexin V (10 µl) and PI (5 µl) were added and incubated for 30 min at room temperature, protected from light, before detection with the Flow cytometer (Beckman, USA). For detection of exposed CALR on the cell membrane surface, treated cells were harvested and blocked with 1.5% bovine serum albumin for 30 min. Then, samples were incubated with primary antibody CALR (Proteintech, 1:200, USA) at room temperature for 40 min followed by secondary antibody PE anti-Rabbit (Abcam, 1:200, USA) for 30 min, and then detect on the flow cytometer. For ATP release detection assay, cell supernatants were collected and then the content of released ATP was detected using the ATP Assay Kit (Applygen, China) according to the manufacturer’s instructions.

### Zebrafish xenograft models

Zebrafish were fed in a 28.5 °C recirculating water system with a standard 12-hour light and dark cycle and fed pellets twice a day. For tumor growth models, zebrafish embryos were mechanically delaminated 2 days after fertilization and anesthetized with 0.15 mg/mL tricaine. 8505 C-NC or siTLR4 cells were stained with 5 µg/mL DiI (Beyotime, C1036) at 37℃ for 30 min. Then, the cells were cleaned twice and resuspended with PBS, microinjected into the perivitelline space (about 500 cells/piece) with the glass capillary needle and grown at 34 °C. At the day 3, the zebrafish were anesthetized and then imaged under the fluorescence microscope. Image pro plus software was used to analyze and calculate the optical density of fluorescence signal.

### Data analysis

All data were represented as the mean ± SD of three independent tests. Each experiment was independently repeated three times. The unpaired Student’s t-tests with GraphPad Prism 7 was utilized to analyze statistical differences between the two groups. *indicated the statistical significance (*P < 0.05, **P < 0.01, ***P < 0.001).

## Results

### Identification and enrichment analysis of DE-ICDGs in anaplastic thyroid carcinoma

**Fig. 2:**
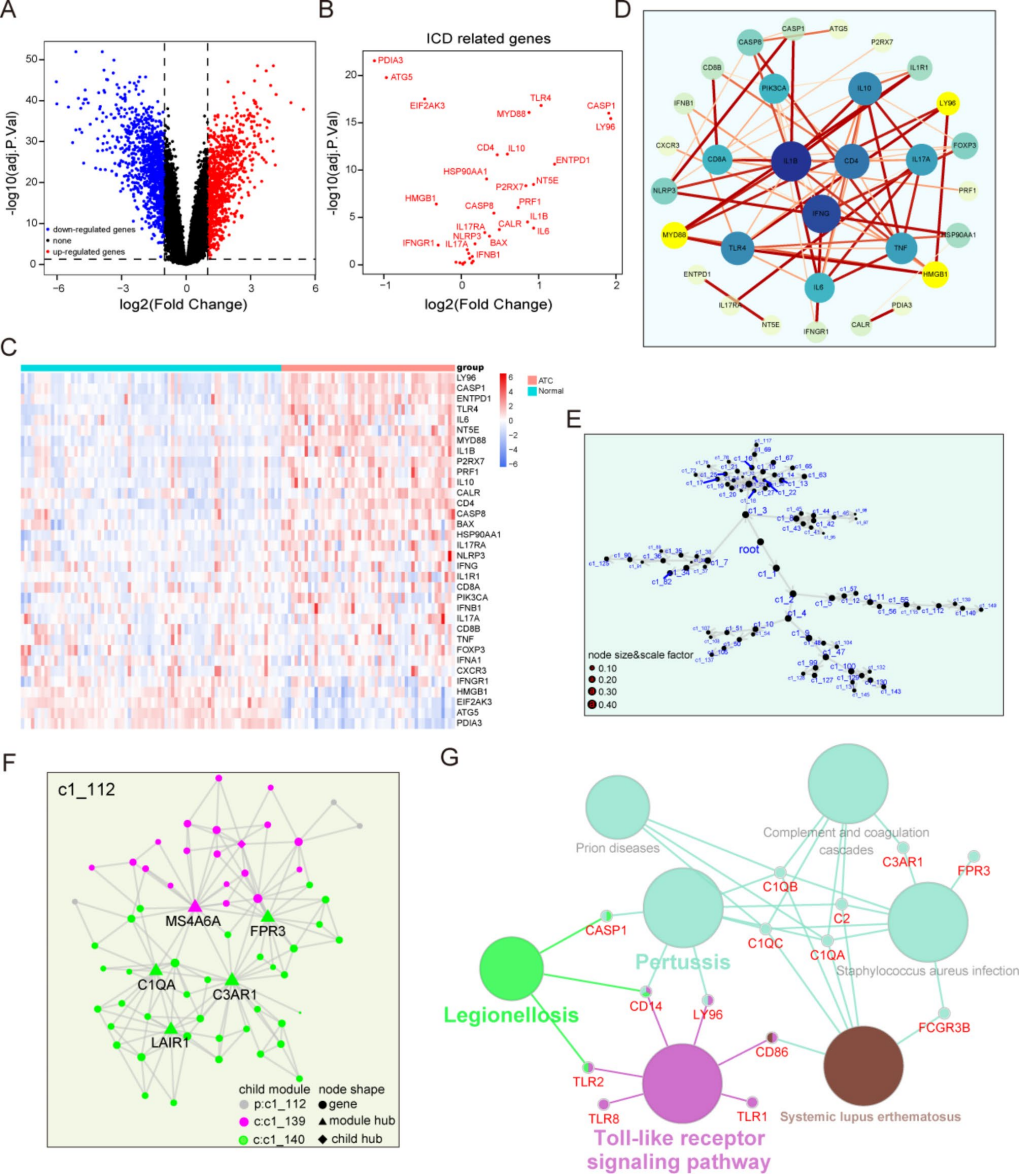
Identification of differential expressed immunogenic cell death genes and network modules analysis in ATC. (**A**) Volcano plot of DEGs identified in the combined dataset according to the fold change and adjusted P value. (**B**) The expression of ICDGs in volcano plot. (**C**) The heatmap of the expression profiles of ICDGs in thyroid cancer samples. (**D**) Protein–protein interactions network of ICDGs. (**E**) The network modules of 1994 DEGs. (**F**) The submodule where DE-ICDGs was located. (**G**) Network analysis diagram of DE-ICDGs and their functions

As stated in our previous study, GSE76039, GSE33630, GSE65144 and GSE29265 were captured and integrated to correct the batch effect while preserving the difference. The combined dataset was analyzed for finding the DE-ICDGs in ATC. As shown in Fig. 2A, a total of 1994 differentially expressed genes were identified from the above dataset. Subsequently, we investigated the expression of ICDGs in normal tissues and ATC (Fig. 2B-C). The analysis of the dataset revealed that a majority of the ICDGs were up-regulated in ATC, while only a few were down-regulated. Notably, LY96, CASP1, ENTPD1, TLR4, and PDIA3 displayed the most significant changes. Furthermore, we constructed a protein–protein interaction (PPI) network with the ICDGs and found that IL1B, IFNG, CD4, IL10 and TLR4 play crucial roles (Fig. 2D). The above results implied that ICDGs may be highly correlated with the progression of ATC.

### The network modules and enrichment analysis of immunogenic cell death genes

The MEGENA analysis were used to construct a gene co-expression network of 1994 DEGs, and further clarify the biomolecular differences associated with the malignant features of ATC. By identifying the interactions of these DEGs, we agglutinated 83 closely linked gene co-expression network modules (Fig. 1E). Then, the co-expression network child module c1_112 which DE-ICDGs were located in was analyzed (Fig. 1F). This module was composed of two gene clusters centered on LAIR1, C1QA, C3AR1, FPR3 and MS4A6A respectively. To investigate the signaling of ATC malignant phenotypes mediated by DE-ICDGs, network enrichment analysis was conducted on all node genes of c1_112 to annotate modules with representative biological function categories (Fig. 1G). Our analysis results demonstrated that these genes can be summarized into 7 specific clusters, and these specific clusters are mainly enriched in 4 biological processes, including Toll-like receptor signaling pathway, legionellosis, pertussis and systemic lupus erthematosus. The results showed that the DE-ICDGs in ATC were highly correlated with changes in these biological processes.

### The immunogenic cell death related score of ATC and underlying signal pathways in different subgroups

**Fig. 3:**
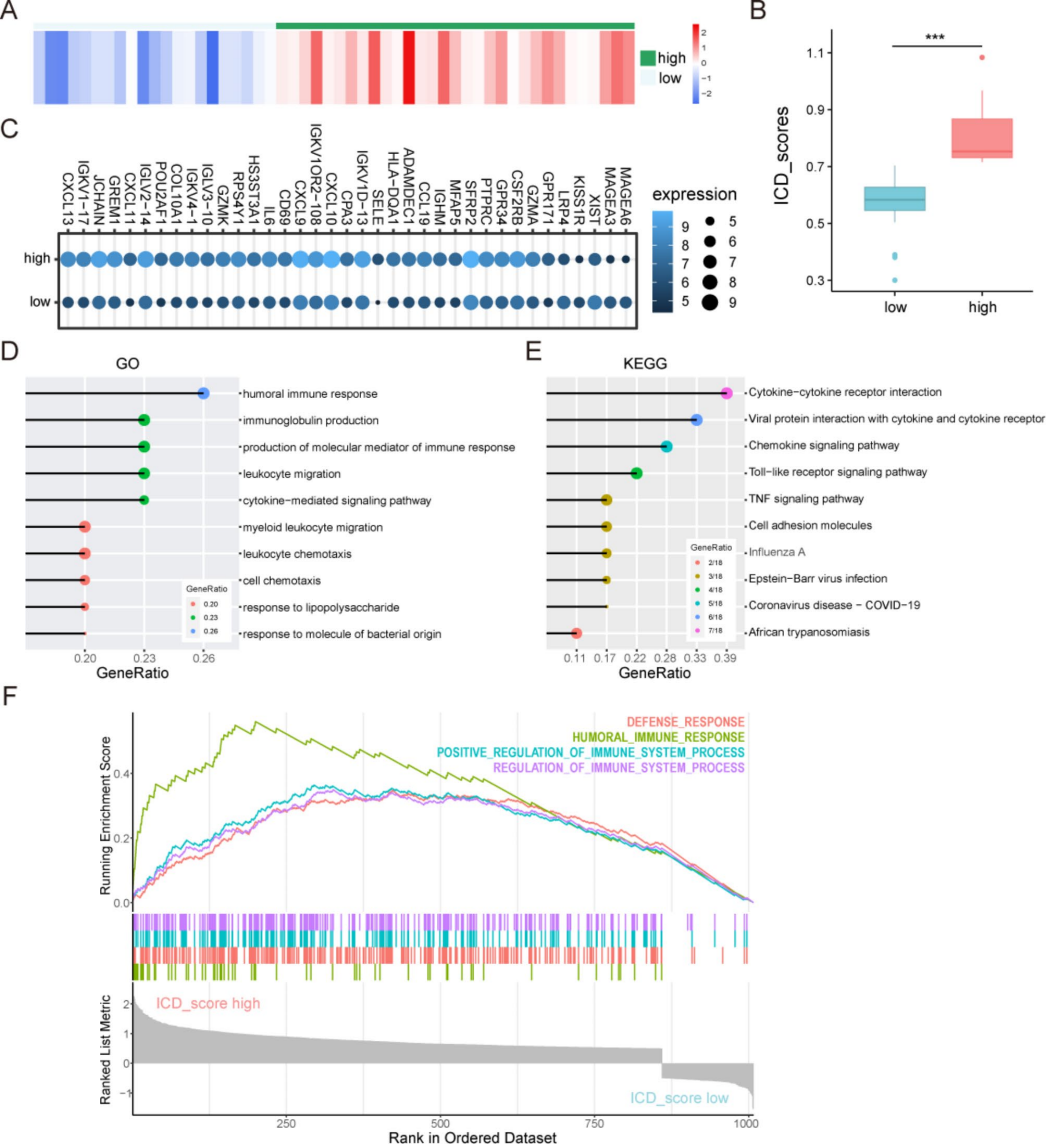
Identification of key differential expressed genes and underlying signal pathways between immunogenic cell death score subgroups. (**A**) The heatmap of ICD scores of ATC samples. (**B**) The boxplot of the different ICD score subgroups. (**C**) The pointplot of DEGs expression between the subgroups. (**D**) GO and (**E**) KEGG analysis of the DEGs. (**F**) The KEGG pathway enrichment of the DEGs by GSEA

Considering the heterogeneity and complexity of individual immunogenic cell death genes expression and subsequent identification of key genes, we performed ssGSEA to quantify the expression of ICDGs in ATC and defined the result as the immunogenic cell death related score (ICD score). Next, ICD score was evaluated for all ATC samples. After calculating the mean value, all patients in the integrated cohort were assigned to the high ICD score and low ICD score groups (*p* < 0.001) (Fig. 3A-B). To explore the differences in the biological behaviors between ICD subgroups, we identified the key DEGs and signal pathways in each subgroups for comprehending the molecular mechanism in regulation of ATC progression (Fig. 3C). Here, we identified 35 upregulated genes and 5 downregulated genes. To assess the functional annotation of these genes in ATC, we conducted GO and KEGG analysis. Our findings revealed that these genes were significantly enriched in activities associated with immunity, such as humoral immune response, immunoglobulin production, production of molecular mediator of immune response and leukocyte migration in the GO analysis (Fig. 3D), as well as cytokine − cytokine receptor interaction, viral protein interaction with cytokine and cytokine receptor, chemokine signaling pathway and Toll − like receptor signaling pathway in KEGG analysis (Fig. 3E). To further identify specifically activated signaling pathways in the high ICD score group, we performed a GSEA comparison between the two groups. As shown in Fig. 3F, enriched gene sets were associated with immune pathways such as defense response, humoral immune response, positive regulation of immune system process and regulation of immune system process. These results indicated that high ICD score was associated with the immune characteristics in ATC.

### Comparison of immune characteristics between the ICD score subgroups

**Fig. 4:**
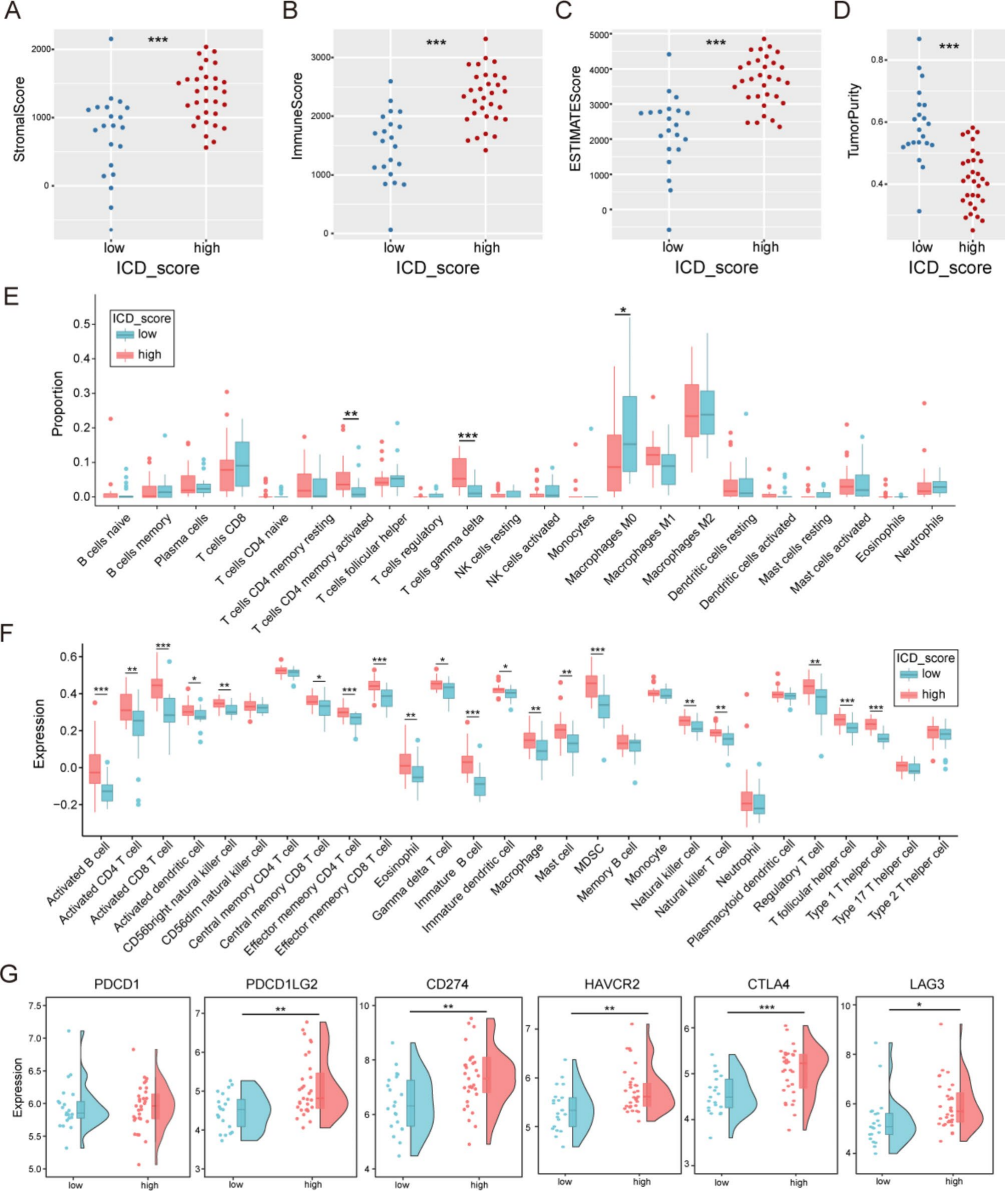
Comparison of immune characteristics between the immunogenic cell death score subgroups. Comparison of the (**A**) stromal score, (**B**) immune score, (**C**) ESTIMATE score, (**D**) tumor purity based on ESTIMATE, (**E**) proportion of immune cells based on CIBERSORT and (**F**) expression of immune cells based on ssGSEA between the high and low ICD score groups. (**G**) Comparison of the expression of immune checkpoints between the ICD score subgroups

In view of the high relationship of ICD score and immune characteristics, ESTIMATE, CIBERSORT, and ssGSEA were performed to comprehend the differences in immunological functions of the subgroups in ATC. ESTIMATE analysis showed that the high ICD score group had a significantly higher immune score, estimate score and stromal score than the low ICD score group, while the tumor purity was lower (*p* < 0.001) (Fig. 4A–D). These results demonstrated that the high ICD score group had higher proportions of immune and stromal cells, and lower proportion of tumor cells. Furthermore, CIBERSORT analysis demonstrated that ICD score has positive correlations with the proportions of T cells CD4 memory activated, T cells gamma delta, and negative correlations with the proportions of macrophages M0 among the 22 tumor-infiltrating lymphocyte types (*p* < 0.05) (Fig. 4E). Simultaneously, ssGSEA analysis showed 28 immune cell subtypes (especially activated B cells, activated CD8 T cells, effector memory CD4 T cell, effector memory CD8 T cell, immature B cell, MDSC, T follicular helper cell and type 1 T helper cell) to be highly expressed in the high ICD score group (*p* < 0.05) (Fig. 4F). We further compared the expression of immune checkpoint molecules in the two groups (*p* < 0.05) (Fig. 4G). According to the data, the mRNA expression of several immune checkpoint molecules revealed that PDCD1LG2, CD274, HAVCR2, CTLA4 and LAG3 were upregulated in the high ICD score group, but there was no significant difference in PDCD1. These results suggested that ICD score had inalienable internal relationship with the immune characteristics of ATC, and high ICD score indicated extensive alterations in the expression of immune checkpoint molecules.

### Identification and validation of key immunogenic cell death genes

**Fig. 5:**
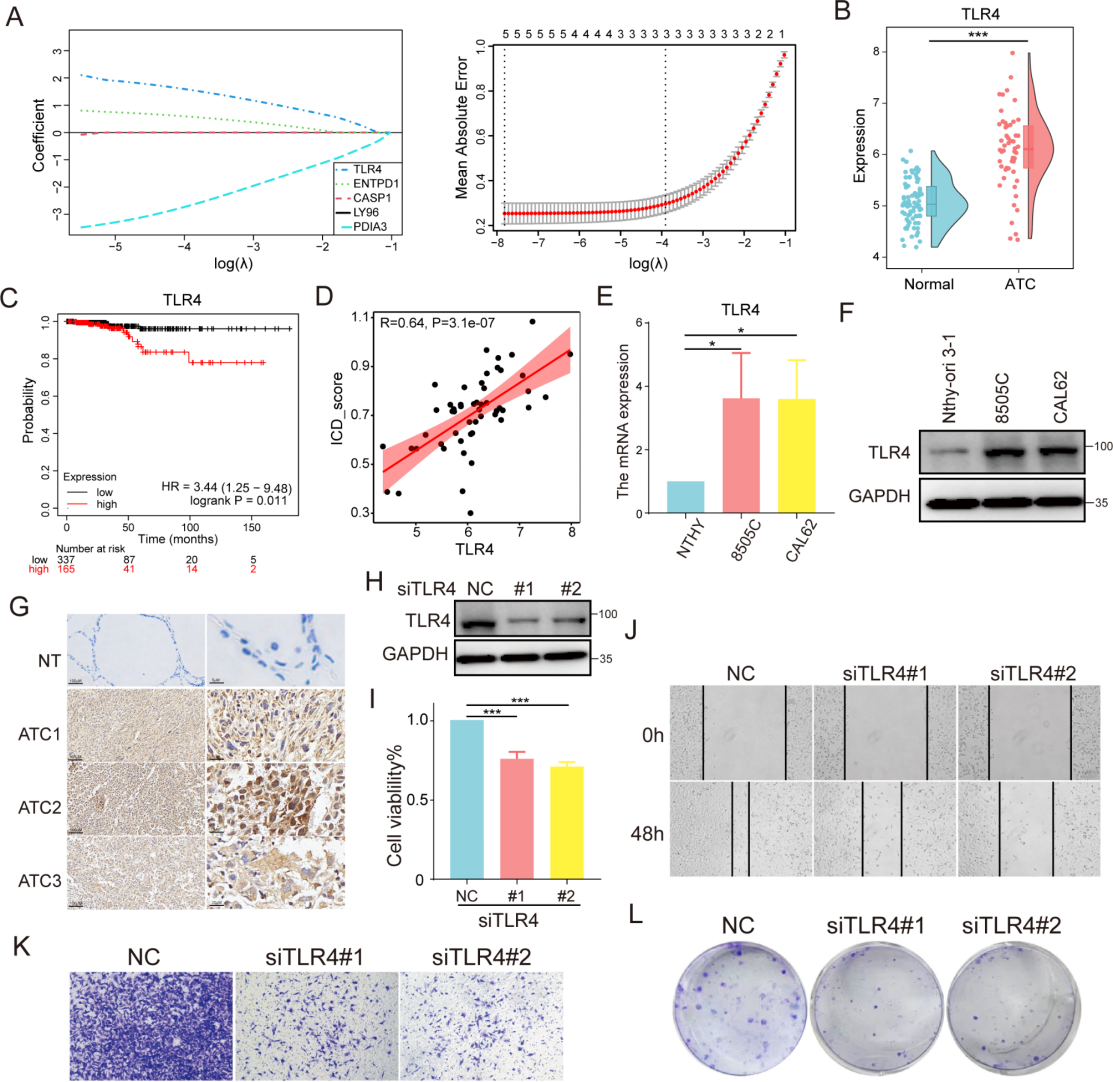
TLR4 regulated the malignant characteristics of ATC. (**A**) The cross-validation applied to adjust parameter selection in the LASSO model. (**B**) The expression of TLR4 between ATC and normal tissues. (**C**) The overall survival of patients with thyroid carcinoma based on TLR4 expression by Kaplan-Meier plotter. (**D**) Correlation between TLR4 expression and ICD score in ATC samples. (**E**) The mRNA and (**F**) protein level of TLR4 in different ATC cell lines (8505 C and CAL62) and normal thyroid cell lines (Nthy-ori 3 − 1, NTHY). (**G**) IHC staining of TLR4 in ATC samples and normal tissue. (**H**) Silencing effect of siRNA-TLR4. The effect of siTLR4 on (**I**) the proliferation for 48 h measured by CCK-8, (**J**) migration ability for 48 h measured by wound-healing assay, (**K**) invasive ability for 48 h measured by transwell assay and (**L**) colony formation ability for two weeks in 8505 C cells

We analyzed the DE-ICDGs in ATC, so that the crucial ICD genes driving the malignant progression of ATC could be identified. The coefficients of 5 genes (TLR4, ENTPD1, LY96, CASP1 and PDIA3) were found to be non-zero by the Lasso-logistic regression model (Fig. 5A). TLR4 with the largest coefficient, was found to be significantly upregulated in ATC and was associated with poor prognosis in patients with thyroid cancer (Fig. 5B-C). In addition, the expression of TLR4 was positively correlated with ICD score in ATC samples (Fig. 5D). For validation, we first detected the expression of TLR4 in ATC and normal thyroid cell lines. As shown in results in Fig. 5E-F, the mRNA and protein levels of TLR4 in ATC was distinctly higher than that in normal (*p* < 0.05). Simultaneously, similar results were obtained by the immunohistochemical staining after determining the expression of TLR4 in ATC and normal thyroid tissues (Fig. 5G). The above results suggested that immunogenic cell death gene TLR4 may act crucial roles in the malignant progression of ATC.

### Downregulation of TLR4 inhibited the malignant phenotypes of ATC

Since TLR4 was upregulated in ATC, whether TLR4 alteration would influence the malignant phenotypes of ATC needs to be further elucidated. Then, siRNA-knockdown ATC model of TLR4 was constructed for subsequent experiments (Fig. 5H). And our data showed that reducing the expression of TLR4 effectively impeded the proliferation of ATC cells (*p* < 0.001) (Fig. 5I). Scratch repair experiments showed that silencing TLR4 reduced the migration ability of ATC cells (Fig. 5J). Next, the invasion ability of ATC cells was significantly weakened after the inhibition of TLR4 (Fig. 5K). Moreover, low levels of TLR4 inhibited the colony formation of ATC cells by clonal formation experiments (Fig. 5L). These findings demonstrated that TLR4 could modulate the malignant progression of ATC. However, the molecular mechanisms which TLR4 involved in remained obscure.

### WGCNA analysis of hub genes related with TLR4 and functional enrichment

**Fig. 6:**
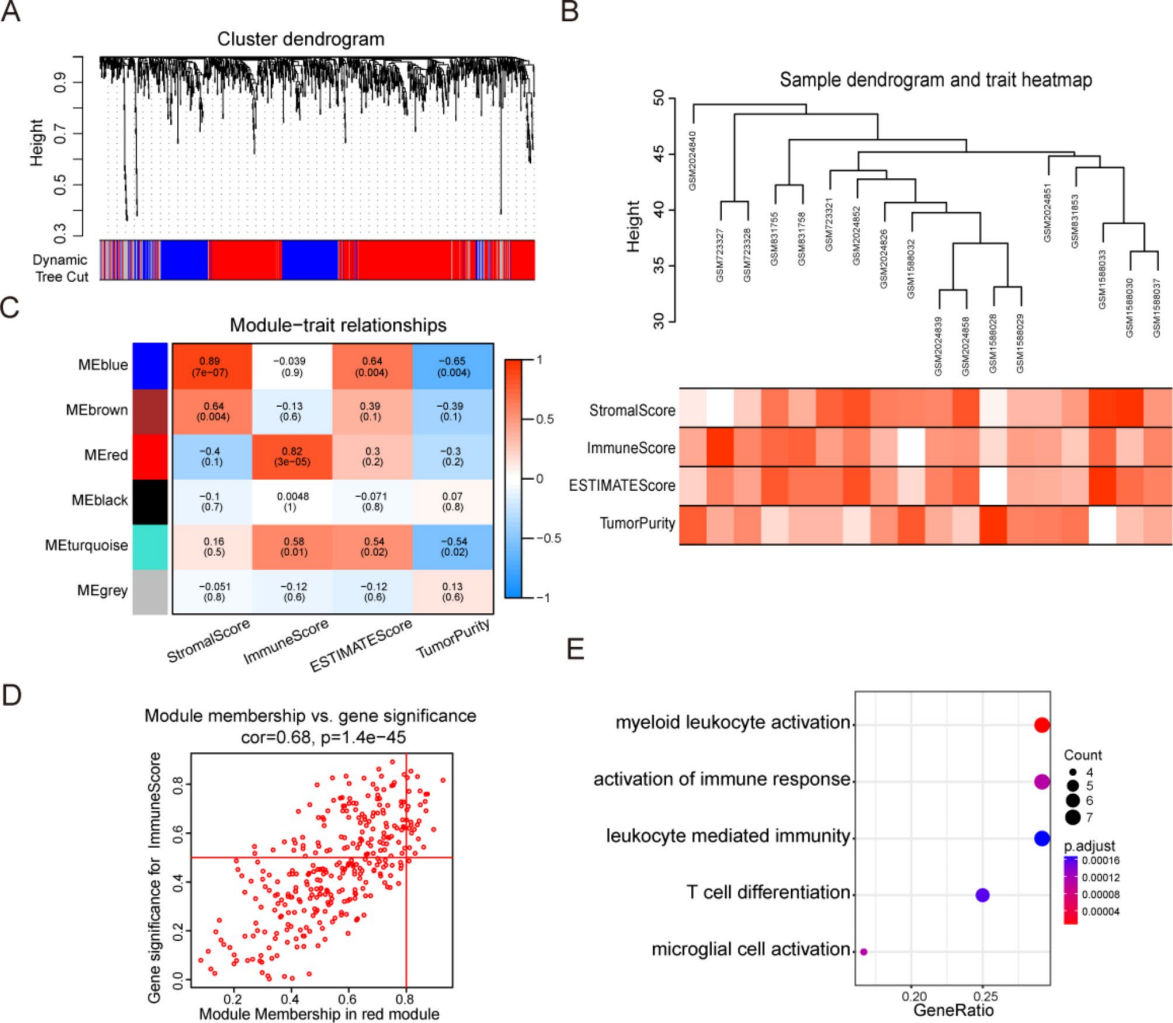
Identification of module genes associated with TLR4 by WGCNA. (**A**) Cluster dendrogram and module colors of ATC. (**B**) Clusters of DEGs based on the dissimilarity measure. (**C**) The correlation heatmap between module eigengenes and ESTIMATE results. (**D**) The scatter plot of red module eigengenes. (**E**) The GO analysis of 26 red module eigengenes

To construct a weighted co-expression network, TLR4-related modules and genes needed to be screened. The ATC samples were divided into two groups based on the TLR4 expression, using the TLR4 expression mean as the reference limit. Weighted gene co-expression network analysis was performed to screen the DEGs. After a series of adjustments for WGCNA parameters, the DEGs were divided into 6 modules by average linkage hierarchical clustering (Fig. 6A-B). We performed the correlation analysis between modules and traits to identify a module associated with TLR4 and immune score (Fig. 6C). The red module contained 326 genes and had the highest correlation with immune score (R = 0.82, P = 3e-05). 26 genes in the red module were selected as hub genes (module absolute membership > 0.8, gene significance > 0.5) (Fig. 6D). To determine the bio-functions of the 26 hub genes, we performed the GO enrichment analysis (Fig. 6E). Myeloid leukocyte activation, activation of immune response, leukocyte mediated immunity, T cell differentiation and microglial cell activation were the most frequently noted pathways in the functional enrichment analysis.

### The inhibition of TLR4 enhanced paclitaxel-induced immunogenic cell death

**Fig. 7:**
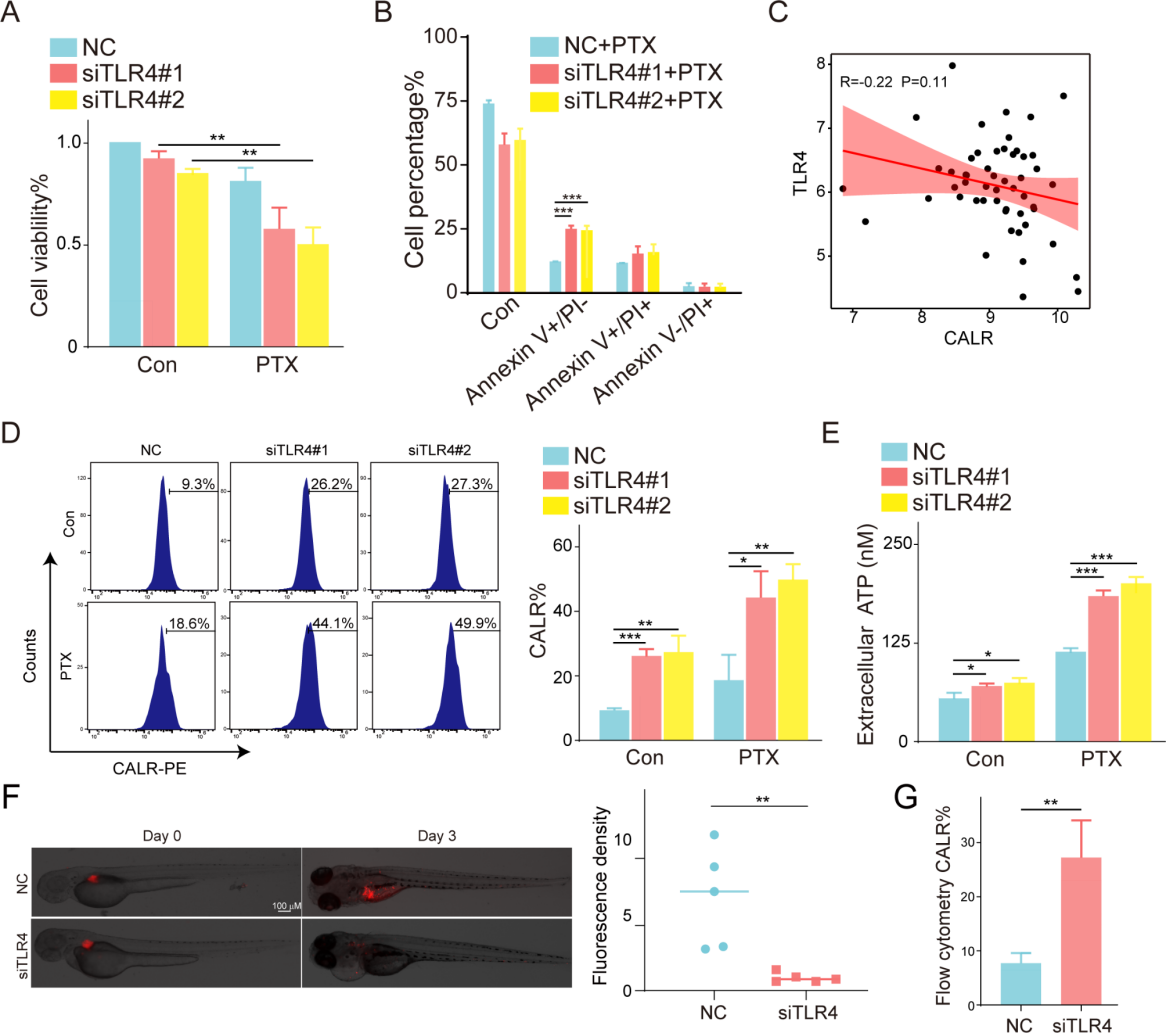
Inhibition of TLR4 enhanced PTX-induced immunogenic cell death. (**A**) The effect of siTLR4 synergistic with PTX (1 nM) on the proliferation for 48 h measured by CCK-8 in 8505 C. (**B**) The apoptosis percentages of 8505 C after the synergistic treatment of siTLR4 and PTX (1 nM) for 48 h measured by flow cytometry. (**C**) Correlation between CALR expression and TLR4 in ATC samples. (**D**) The CALR exposure and (**E**) ATP release of 8505 C after the synergistic treatment of siTLR4 and PTX (1 nM) for 48 h. (**F**) The tumorigenesis of zebrafish in NC and siTLR4 groups. (**G**) The CALR exposure of NC and siTLR4 groups in zebrafish

Moreover, with considering that TLR4 was an important ICD gene and the reported roles in PTX-induced ICD, we first silenced TLR by siRNA transfection for 24 h and then treated with 1 nM PTX for 48 h. The results indicated that the repressed expression of TLR4 could effectively enhance PTX-induced proliferation inhibition and apoptosis of ATC cells (*p* < 0.01) (Fig. 7A-B). Simultaneously, a correlation analysis revealed a negative correlation between the expression of TLR4 and CALR whose exposure was one of the DAMPs in ICD (Fig. 7C). For verification, we observed more CALR exposure on the ATC cell membrane by flow cytometry (Fig. 7D). And the release of ATP was significantly increased in the TLR4 inhibition and PTX synergistic groups (*p* < 0.05) (Fig. 7E). In addition, we also found that silencing TLR4 could significantly reduce the level of CALR and inhibit the tumors growth in zebrafish xenograft models (*p* < 0.05) (Fig. 7F-G). The above contents suggested that silencing TLR4 could synergize with PTX to induce ICD in ATC.

## Discussion

Cancer immunotherapy was to stimulate the immune system to elicit the body’s immune response to the tumor, but tumor immune escape made its clinical efficacy limited [32, 33]. Accumulating evidence clearly underscored that ICD activated the innate immune system and actively modulates cancer immunotherapy [34, 35]. Understanding the molecular signature of ICDGs had the potential to identify straregies to overcome the immune escape “cold tumor”. This knowledge opened up new avenues for enhancing the effectiveness cancer immunotherapy. Despite the considerable efforts to characterize ICD-related genes or ICD-inducers in other cancers, the dynamic profiling of ICDGs in ATC remained far from being established [36, 23, 37–39]. In this study, large cohort data of ATC samples were integrated by us to analyze the signature of ICDGs in ATC. We found that most ICDGs were upregulated in ATC and had close interaction. Next, MEGENA analysis was performed to identify the submodules of ATC gene network that ICDGs belonged to. Preliminary, it was found that they mainly mediated Toll-like receptor signaling pathway, and then affected the malignant characteristics of ATC.

Further, the ICD score model was established for classifying ATC samples, with significant differences between subgroups.

Obviously, these genes were found to be strongly linked to immune response, immune system regulation, and cytokine signaling, as verified through functional enrichment analysis. Following this, a comparison of the immune characteristics was conducted between two subgroups. The tumor purity was lower in the high ICD score group because of the massive infiltration of immune cells and stromal cells in its tumor microenvironment. These overabundant immune cells had the largest difference in the proportion of intermediate-class T cell subtypes, including activated CD8 T cells, effector memory CD4 T cell, effector memory CD8 T cell, immature B cell, T follicular helper cell and type 1 T helper cell. It was implied that ICDGs was associated the dysfunction of T cell in ATC. T cell dysfunction was one of the pivotal mechanisms of tumor immune escape [40]. During tumor development, T cell dysfunction/exhaustion occurred and various checkpoint inhibitory receptors were upregulated, thus limiting the survival and functions of T cells [41, 42]. Emerging evidences had shown that the increased immune checkpoint molecules were described as the hallmarks of T cell exhaustion. Indeed, in our study, the expression of PDCD1, PDCD1LG2, CD274, HAVCR2, CTLA4 and LAG3 were upregulated in the high ICD score group. These results suggested that high ICD score represented extensive alterations in immune characteristics of ATC.

Creatively, the Lasso-logistic model was performed to analyze ICDGs, and we discovered a signature of 5 ICDGs in ATC. This findings helped us gain a deeper our understanding of the polymorphism of ICD in cancer. These ICDGs (TLR4, ENTPD1, LY96, CASP1 and PDIA3) were rarely reported in ATC. Notably, the expression of ENTPD1 in ATC was higher than that in normal tissues, suggesting its significance in regulating metabolic pathway and acting important roles in thyroid cancer [43, 44]; LY96 was highly associated with increased intratumoral lymphocytic infiltration in oncocytic PDTC [45]; CASP1 was identified as the most promising biomarker for the diagnosis or treatment of PTC and involved in molecular changes in brain metastatic PTC [46, 47]; patients with low PDIA3 expression had lower etiological specific survival than those with high PDIA3 expression in PTC [48]. These researches further enhanced the authenticity and credibility of our analytical strategies. Even if we did not explore these genes in depth, their pivotal roles in the progression of ATC could not be ignored.

Under physiological conditions, TLR4 was a critical regulator of inflammatory responses and tissue regeneration, thus maintaining tissue homeostasis [49]. Notably, accumulated evidence showed abnormal expression of TLR4 signaling in several tumors. Indeed, functional expression of TLRs in tumor cells was actively involved in tumor survival and progression, immune escape and apoptotic resistance [50]. Nevertheless, there have been inconsistences in impact of TLR4 activation on cancer cells across various cancer models. TLR4-induced secretion of IL-1β shaped the immunosuppressive microenvironment of pancreatic cancer [51]. MAPK/ERK signal-dependent TLR4 expression promoted the survival signaling and stemness, thus facilitating the PTC progression with BRAF-V600E mutations [52, 53]. Conversely, the research on pituitary epithelial neoplasms had found that activation of TLR4 impeded tumor cell growth [54]. Likewise, we found that the up-regulation of TLR4 was positively correlated with ICD score in ATC, and significantly correlated with the poor prognosis of thyroid cancer patients. It was curious about the ambiguous role of TLR4 in ATC progression.

In our study, we verified that both mRNA and protein levels of TLR4 were upregulated in ATC cell lines. After the silence of TLR4 expression, the ability of proliferation, metastasis, invasion and clone formation were significantly inhibited.

These results demonstrated that TLR4 was a crucial driver of ATC malignant phenotypes. Additionally, the WGCNA analysis revealed that the red module associated with TLR4 exhibited the strongest correlation with immune score, providing further insights into the potential molecular mechanisms of TLR4 in ATC. And enrichment analysis had found the genes related TLR4 involved in myeloid leukocyte activation, activation of immune response, leukocyte mediated immunity, T cell differentiation and microglial cell activation pathways. Nonetheless, more work was urgent to explain how TLR4 regulates these immune-related pathways in ATC. Intriguingly, we found that reducing TLR4 expression significantly enhanced PTX-induced proliferation inhibition and apoptosis, as well as increased CALR exposure and ATP release. These release of DAMPs were ICD-specific, although this was in contrast to the effect of TLR4 reported in the literature [13]. In addition, whether TLR4 regulated ICD based on immune or other signals required further validation.

In conclusion, we highlighted the first dynamic panorama of ICDGs and the immunological characteristics in ATC. These results might be facilitated for ICD-based immunotherapy interventions in ATC patients. We also identified and verified TLR4, the pivotal ICD-related gene driving the progression of ATC malignancy, which held great prospect and value for inducing ICD and improving ATC treatment.

### Electronic supplementary material

Below is the link to the electronic supplementary material.

**Supplementary Material 1**: The row bands of western blot

## Data Availability

The microarray datasets (GSE65144, GSE33630, GSE29265 and GSE76039) were gotten from the GEO database (https://www.ncbi.nlm.nih.gov/geo/). In addition, all data for this study are available from the corresponding author upon reasonable request.
