# Supplementary material for: Immunological characteristics of immunogenic cell death genes and malignant progression driving roles of TLR4 in anaplastic thyroid carcinoma
Source: BMC Cancer. 2023 Nov 21;23:1131. doi: 10.1186/s12885-023-11647-y (PMC10664293; doi:10.1186/s12885-023-11647-y)
Supplement: Supplementary file 1 — Supplementary Material 1: The row bands of western blot [file 12885_2023_11647_MOESM1_ESM.pdf]

Immunological characteristics of immunogenic cell death genes and malignant progression driving roles of TLR4 in anaplastic thyroid carcinoma

Tong Xu[1], Chaozhuang Zhu[2], Feifeng Song[1], Wanli Zhang[2], Mengnan Yuan[1] Zongfu Pan[1,3], and Ping Huang[1,3,*]

Raw blot:

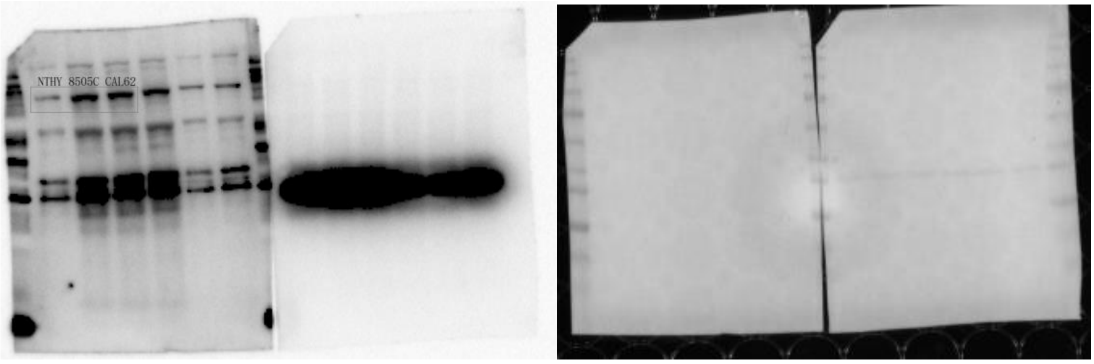

Fig5F-TLR4 The protein level of TLR4 in different ATC cell lines (8505C and CAL62) and normal thyroid cell lines (Nthy-ori 3-1, NTHY).

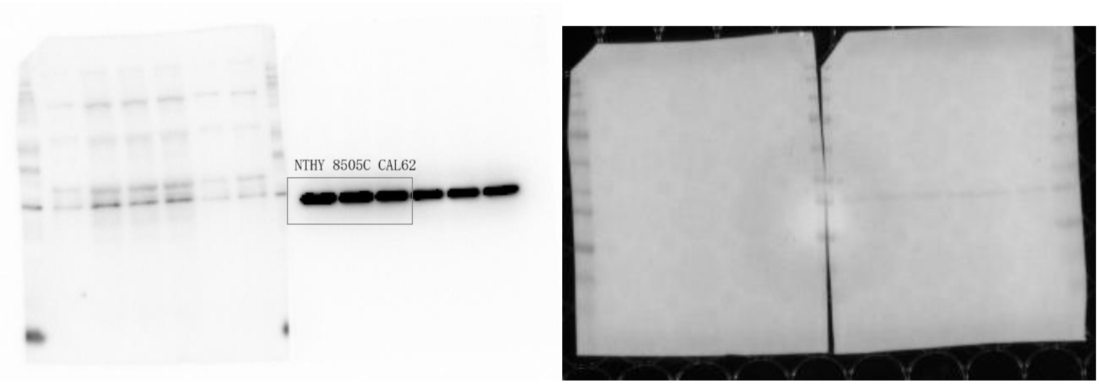

Fig5F-GAPDH The protein level of TLR4 in different ATC cell lines (8505C and CAL62) and normal thyroid cell lines (Nthy-ori 3-1, NTHY).

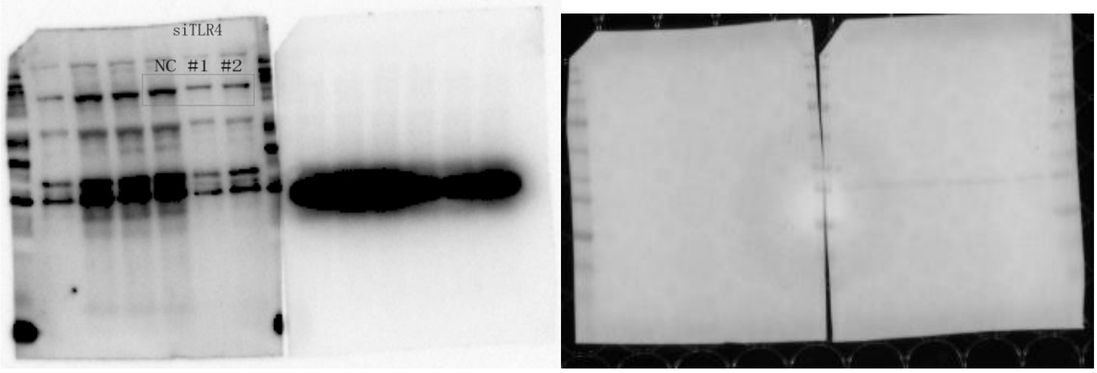

Fig5H-TLR4 The silencing effect of siRNA-TLR4.

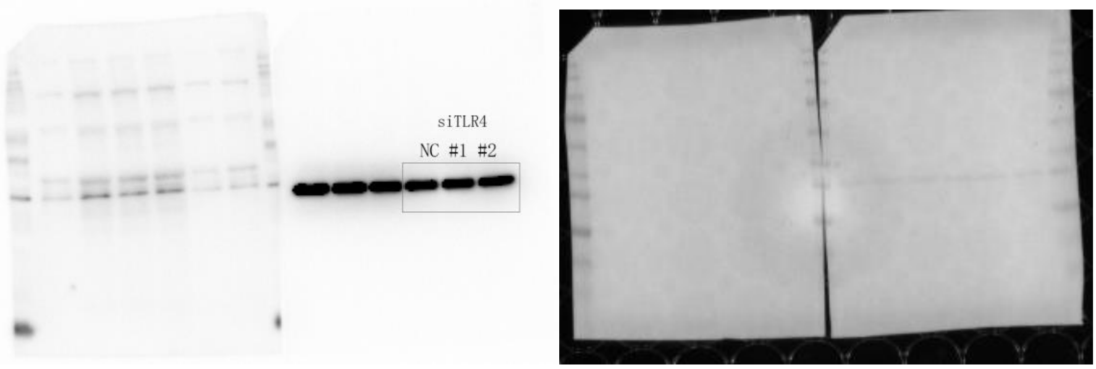

Fig5H-GAPDH The silencing effect of siRNA-TLR4.
